# SG-APSIC1178: Factors influencing COVID-19 prevention practices among healthcare personnel in Rajavithi Hospital

**DOI:** 10.1017/ash.2023.25

**Published:** 2023-03-16

**Authors:** Kampong Kamnon

**Affiliations:** Rajavithi Hospital, Bangkok, Thailand and Daranee Sornsuwan, Rajavithi Hospital, Bangkok, Thailand

## Abstract

**Objectives:** To determine the factors influencing COVID-19 prevention practices among healthcare personnel. **Methods:** The sample consisted of healthcare personnel working in the emergency department, inpatient wards, and the outpatient department in 250 Rajavithi hospitals selected using a purposive sampling method. Data were collected using questionnaires that were validated by 5 experts and had a content validity index of 0.83. The reliability of the questionnaires was 0.91. Data were analyzed using descriptive statistics and multiple regression. **Results:** Study participants had good attitudes toward behaviors, subjective norms, perceived behavioral control, and intention to prevent COVID-19. In addition, perceived behavioral control was the only factor that statistically predicted intention to perform COVID-19 infection prevention and may explain 25.6% of the variability of intention (*P* < .001). **Conclusions:** Based on the results of this study, relevant authorities, including wards and infection control units, should support perceived behavioral control among registered nurses to encourage COVID-19 prevention practices.

